# Rainbow trout integrated response after recovery from short-term acute hypoxia

**DOI:** 10.3389/fphys.2022.1021927

**Published:** 2022-10-21

**Authors:** Irene García-Meilán, Lluis Tort, Ali Reza Khansari

**Affiliations:** ^1^ Departament de Biologia Cel·lular, Facultat de Biologia, Universitat de Barcelona, Barcelona, Spain; ^2^ Department of Cell Biology, Physiology and Immunology, Faculty of Biosciences, Universitat Autònoma de Barcelona, Bellaterra, Spain

**Keywords:** hypoxia, stress response, cortisol, gene transcript expression, brain, head kidney, skin, rainbow trout

## Abstract

Overcoming a stress situation, such as hypoxia episodes, which involve an allostatic load, will depend on the ability of fish to modulate physiological and biochemical systems to maintain homeostasis. The aim of the study was to determine the integrated stress response after acute hypoxia of the rainbow trout considering the different elements and areas of the stress response: systemic and mucosal, local and global, and from the systemic hypothalamic–pituitary–interrenal axis to skin mucosa. For this purpose, trout were subjected to acute hypoxia (dissolved O_2_ down to 2 mg/L) for 1 h and then recovered and sampled at 1, 6, and 24 h after reoxygenation. Physiological responses were significantly affected by hypoxic stress and their interaction with time after the challenge, being significant for plasma lactate and cortisol levels, in both plasma and skin mucus. At the central brain level, only *trh* expression was modulated 1 h after hypoxia which indicates that brain function is not heavily affected by this particular stress. Unlike the brain, the head kidney and skin were more affected by hypoxia and reoxygenation. In the head kidney, an upregulation in the expression of most of the genes studied (*gr*, *il1β*, *il6*, *tgfβ1*, *lysozyme*, *caspase 3*, *enolase*, *hif-1*, *myoglobin*, *sod2*, *gpx*, *gst*, and *gsr*) took place 6 h after recovery, whereas only *hsp70* and *il10* were upregulated after 1 h. On the contrary, in the skin, most of the analyzed genes showed a higher upregulation during 1 h after stress suggesting that, in the skin, a local response took place as soon as the stressor was detected, thus indicating the importance of the skin in the building of a stress response, whereas the interrenal tissue participated in a later time point to help prevent further alteration at the central level. The present results also show that, even though the stressor is a physical/environmental stressor, all components of the biological systems participate in the regulation of the response process and the recovery process, including neuroendocrine, metabolism, and immunity.

## Introduction

Overcoming a stress situation, such as hypoxia episodes, will depend on the ability of fish to modulate physiological and biochemical systems, such as changes in energetics and metabolism, cardio-respiratory rhythms, and other adjustments including behavior (Ton et al., 2003; Dawood et al., 2021) to maintain homeostasis.

When stress is perceived, it triggers the hypothalamic–sympathetic–chromaffin (HSC) axis that activates a quick response involving adrenaline release to modulate the cardio-respiratory system and providing glucose as an energy source (Eiden 2013; Kvetnansky et al., 2013), and later, the hypothalamic–pituitary–interrenal (HPI) axis plays a central role in acclimation to stress by cortisol release (Mommsen et al., 1999; Gorissen and Flik, 2016). Moreover, an integrative response that involves all regulatory systems (neural, endocrine, and immune) is activated. In this sense, some mediators, such as stress hormones or specific inflammatory cytokines, become key interaction molecules of bidirectional immunoendocrine regulation (Engelsma et al., 2002; Tort, 2011).

While the lack of oxygen rarely occurs in terrestrial animals, in the aquatic environment, oxygen can become a limiting factor. Hypoxia, the condition where dissolved oxygen drops from the normoxic 8 mg O_2_/L to low levels such as 2.8 mg O_2_/L (Diaz and Rosenberg, 1995), has been often observed. Hypoxic episodes may occur due to different reasons, such as eutrophication caused by excessive input of nutrients or temperature increase, which may become more common due to global warming in natural environments. Although some fish groups have evolved to survive in environments with low or variable levels of dissolved oxygen, these fluctuations can lead to temporary hypoxia, becoming an abiotic stressor for fish. Thus, different responses after hypoxia stress have been found in fish, like suppressive effects on the immune system and expression of the stress-immune- and hypoxia-related genes (Welker et al., 2007; Tiedke et al., 2014; He et al., 2019; Mu et al., 2020; Wang Q. et al., 2021; Schäfer et al., 2021; Hung et al., 2022), negative effects on growth (Li et al., 2017), feed utilization (Sheng et al., 2019), respiration, and osmoregulation (Sun J.-L. et al., 2020), among others. As mentioned previously, hypoxia stress induces the activation of the HPI axis, and it has been shown that after hypoxic situations several immune indicators both from humoral and cellular responses are implicated (Welker et al., 2007). Moreover, when fish are subjected to hypoxia, not only central organs such as the brain and head kidney (HK) are concerned, but the skin may also play an important role, as it is a key surface for sensory environmental perception including hypoxia, among other functions such as communication, respiration, osmoregulation, and locomotion (Sanahuja and Ibarz, 2015). It should be also noted that this non-keratinized multi-layered integument produces skin mucus, a matrix that includes relevant molecules that play a role as mediators of the stress reactions, such as cortisol, glucose, or immune peptides. In addition, it has been demonstrated that one of these main mediators of the stress response, cortisol, shows a consistent correlation in its concentration between plasma and mucus (Declerq et al., 2016; Guardiola et al., 2016; De Mercado et al., 2018; Fernández-Alacid et al., 2018; Carbajal et al., 2019).

Our hypothesis in this work is that acute stress due to hypoxia not only affects oxygen distribution systems and induces metabolic adjustments but also involves effects on the immune, energetics, and antioxidant systems, and for this reason, we have chosen not only the current physiological and metabolic indicators but also representative genes of immune, endocrine, and neural systems in key organs such as the brain, HK, and skin. Consequently, the objective of this work was to determine the integrated stress response after acute hypoxia of rainbow trout considering the different elements and areas of the stress response: systemic and mucosal, local, and global, with the aim of characterizing the different components of this integrated response, from the systemic HPI axis to skin mucosa. In this sense, hematological and physiological indicators, including hematocrit, plasma glucose and lactate, and plasma and skin mucus cortisol, were determined in rainbow trout, together with the transcript-level pattern of specific genes related with stress and hypoxia.

## Materials and methods

### Experimental design and sampling

The juveniles of rainbow trout were distributed randomly in duplicate tanks of 300 L per condition (4 tanks) and acclimatized to the facilities of the AQUAB-lab group (Universitat Autònoma de Barcelona, UAB). The fish were fed *ad libitum* with a commercial diet (Skretting) and subjected to a photoperiod of 12L:12D and 18°C in a closed recirculation system. After the acclimatization period, the trout, with a final body weight of 80.8 ± 3.5 g, were subjected to a stress challenge by acute hypoxia for 1 h and then were allowed to recover to the normal rearing condition. There were two experimental groups by duplicate: fish under normoxia (norm) and fish sampled after a hypoxia challenge and reoxygenation at different times: 1, 6, and 24 h post-hypoxia. To carry it out, air pumps were removed from water, and N_2_ was injected in the system by bubbling, reducing oxygen levels in water from 8 mg/L to 2 mg/L. The oxygen levels of the water were continuously monitored during the experiment using an oximeter (Thermo Scientific, Madrid). After the challenge, eight fish for each treatment (4 fish/tank) and time were euthanized with an overdose of phenoxyethanol, measured, weighed, and samples of blood, skin, and mucus were quickly collected, and the brain, HK, and skin were excised. All animal-handling procedures carried out in this study complied with the Guidelines of the European Union Council directive (EU 2010/63) and were approved by the Ethics and Animal Care Committee of the Universitat Autònoma de Barcelona (permit numbers OH4218_4219 and DAAM 11251), following the regulations and procedures established by the Spanish and Catalan governments.

### Hematocrit, biochemical, and hormonal analyses

Blood samples were collected by puncturing the caudal vein using a heparinized syringe, and an aliquot was directly used to determine the hematocrit. After that, the plasma was isolated by centrifugation (1500 x g, 15 min, 4°C) and stored at −20°C until the analysis. Plasma glucose and lactate concentrations were measured with commercial kits (LO-POD glucose and LO-POD lactate, Spinreact, Spain), whose protocols have been standardized and adapted to microplate dimensions. All tests were carried out in duplicate and were expressed according to the commercial standards used.

Skin mucus was sampled according to Xu et al. (2013). Briefly, skin mucus was collected by gently scraping the fish skin into a 15-ml Falcon tube and homogenized using a syringe (by taking and blowing out the whole content several times). Samples were first centrifuged for 10 min at 1,500 rpm, and the supernatant was transferred to an Eppendorf tube that was centrifuged again for 10 min 10,000 g at 4°C, and then the samples were stored at −20°C until further analysis. Skin tissue samples (upper-lateral line area behind the dorsal fin, left side, and roughly of the same size) were carefully taken to avoid muscle contamination. The brain and HK were also sampled and immediately frozen in liquid nitrogen and stored at −80°C for further assays.

Plasma and skin mucus cortisol levels were analyzed by radioimmunoassay (RIA) as previously described by Rotllant et al. (2006). Briefly, samples of diluted denatured plasma or skin mucus were used, and cortisol was detected using a 1:4,500 final dilution of antibody purchased from MO Bio-medical LLC (United States).

### RNA extraction and cDNA synthesis

The total RNA was isolated from about 100 mg of tissue (brain, HK, and skin) in 1 ml of TRI Reagent solution^®^ (Sigma Aldrich, United States) following the manufacturer’s instructions. The concentration and purity of RNA were determined at 260 nm with an A260/A280 purity ratio using a NanoDrop 2000 Spectrophotometer (Thermo Fisher Scientific Inc. United States). The cDNA of each sample was synthesized from 2 μg of RNA using the High-Capacity cDNA Reverse Transcription Kit (Applied Biosystems, United States) following the manufacturer’s instructions, using first oligo (dT) 15 and random hexamers.

### Real-time quantitative PCR

Transcript levels were analyzed using real-time quantitative PCR (RT-qPCR) in a CFX Touch™ Real-Time PCR Detection System (Bio-Rad, United States). In the brain, HK, and skin, genes related to inflammation and innate responses (*il6*, *il10*, *il1β*, *tgfβ1*, *lysozyme*, and *caspase3*), antioxidant genes (*sod2*, *cat*, *gpx*, *gsr*, and *gst*), stress-related genes (*hsp70*, *crh*, *trh*, and *gr*), and genes related to hypoxia (*hif-1*, *enolase*, and *myoglobin*) were determined using *βactin* and *18S* as reference genes. Briefly, specific primers used are listed in table 1. Primers were designed using Primer-Blast. The primer secondary structure and annealing specificity were checked using OligoAnalyzer software (version 3.1) and Primer-Blast software, respectively. The undesirable PCR product appearance was previously verified by a single peak in the melting curve for each primer set. The primer amplification efficiency was determined in all tissues included in our study. Real-time PCR reactions were performed with iTaq Universal SYBR Green supermix (Bio-Rad Laboratories) using 1:20 cDNA dilution made for genes of interest. Primers for all genes were used at a final concentration of 500 nM. The thermal conditions used were 3 min at 95°C of pre-incubation followed by 40 cycles at 95°C for 30 s and 60°C for 30 s. All the reactions were performed in duplicate using CFX384 Touch Real-Time PCR Detection System (Bio-Rad Laboratories). The efficiency of the amplicon and the size of the product are presented in Table 1. Quantification was performed according to the BIORAD formula using *18S* and *βactin* as reference genes and efficiency of each primer set obtained for each tissue. Values for the experimental condition were expressed as normalized relative expression against those of the housekeeping genes *18s* and *βactin*.

**TABLE 1:** Primers used for transcript-level analysis in rainbow trout.

Gene	Official name	Accession number	Sequence 5′-3′	Efficiency	Product length
*18S*	Ribosomal protein S18	XR_005034822.1	**FW**:TGAGCAATAACAGGTCTGTG	1.96	212
			**RV:**GGGCAGGGACTTAATCAA		
*Β-actin*	Beta actin	XM_036972332.1	**FW**:GGACTTTGAGCAGGAGATGG	1.95	186
			**RV:**ATGATGGAGTTGTAGGTGGTCT		
*Caspase 3*	Caspase 3	XM_021561577.2	**FW**:GAGATGGCCTCCAAGAAGATAGAA	1.96	91
			**RV:**ACCGCATGTACGCATCATCA		
*Cat*	Catalase	XM_021568213.2	**FW**:GCAGTGCCTTTTTGGGTTAGT	1.82	175
			**RV:**ACCAAACCACAACTCTTCAGTG		
*Crh*	Corticotrophin-releasing hormone	NM_001124286.1	**FW**:AAGTTGGGAACATCAGCCG	1.89	127
			**RV:**ATCTGTCGGAGCATGTGGAAC		
*Enolase*	Enolase	XM_036988980.1	**FW**:CAAAGGTGTCTCAAAAGCCG	2.00	73
			**RV:**GTTGACGTTCTGCCGTACAA		
*Gpx*	Glutathione peroxidase	XM_021569971.2	**FW**:ATTCCCCTCCGATGACTCCA	1.94	155
			**RV:**TGGTCAGGAACCTTCTGCTG		
*Gr*	Glucocorticoid receptor	NM_001124730.1	**FW**:GCTGCTTTCCTTTCCTCCCT	1.99	149
			**RV:**GAGACACCAGCCTCCAGAAG		
*Gsr*	Glutathione reductase	XM_021561547.2	**FW**:CGATTGCTTCCACCCTCTTAC	1.89	196
			**RV:**AGCCGACATTGACACAGGTA		
*Gst*	Glutathione-S-transferase	XM_021561454.2	**FW**:TATTGTGGGCTAATGTGTAAGAT	1.95	215
			**RV:**CCCTGAAGAGCTTTGTCG		
*hif-1a*	Hypoxia-inducible factor 1 alpha	NM_001124288.1	**FW**:TTCTCTGTGCTCTTCTGTGCG	1.96	161
			**RV:**TGAGTAAGGAAGCAGGGCAA		
*hsp70*	Heat shock protein 70	NM_001124228.1	**FW**:ATTCTGAACGTAGCAGCGGT	1.99	158
			**RV:**GCCATCTTCTCCCTCTGTGC		
*il1β*	Interleukin 1 beta	XM_036979104.1	**FW**:CTGAAGCCAGACCTGTAGCC	2.01	100
			**RV:**GCAACCTCCTCTAGGTGCAG		
*il6*	Interleukin 6	NM_001124657.1	**FW**:GAGTTTCAGAAGCCCGTGGA	2.09	149
			**RV:**AGCTGGTACACTTGCAGACC		
*il10*	Interleukin10	NM_00124599.1	**FW**:CGACTTTAAATCTCCCATCGAC	1.89	70
			**RV:**GCATTGGACGATCTCTTTCTT		
*Lys*	Lysozyme	XM_021601582.2	**FW**:TGCCTGTCAAAATGGGAGTC	1.99	152
			**RV:**CAGCGGATACCACAGACGTT		
*Myoglobin*	Myoglobin	NM_001171862.1	**FW**:AACAAACACGGAGGACTGGTT	1.97	85
			**RV:**CGATGCCTGCGAACTTAGGG		
*sod2*	Superoxide dismutase	XM_021612540.2	**FW**:TCCCTGACCTGACCTACGAC	1.90	201
			**RV:**GGCCTCCTCCATTAAACCTC		
*tgfβ1*	Transforming-growth factor beta 1	XM_036961610.1	**FW**:GGGCGACAGCAGACGATACC	1.97	102
			**RV:**GGCCTCCTCCATTAAACCTC		
*Trh*	Thyroid-releasing hormone	XM_021566749.2	**FW**:CTGCTCCGCTCCATTCTCAA	2.08	142
			**RV:**TCCCAGGGTGTTGCCTTTTT		

### Statistical analysis

Statistical Package for Social Science (SPSS, v25) software was used for the analysis. The generalized linear model (GzLM) was utilized considering the hypoxia and time dynamics as a two between-subjects factor. This model is more a flexible statistical tool than the standard general linear model (GLM) in terms of types of distribution and different covariance structures of the repeated measures, and does not require homogeneity of variance, and it admits missing values. After the main analysis, appropriate pair-wise comparisons were carried out. Differences in all data were considered statistically significant if p-values < 0.05 among groups.

## Results

**FIGURE 1:**
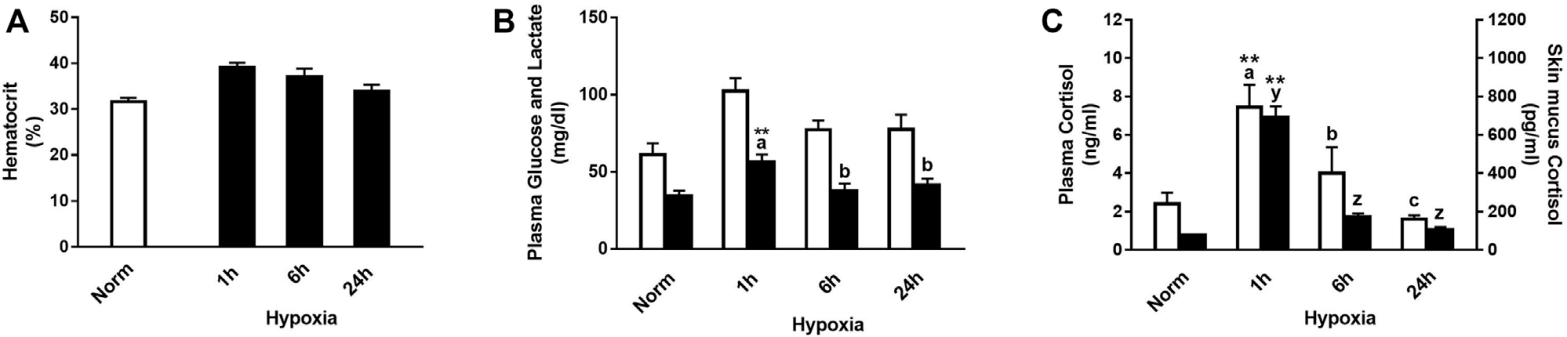
**(A)** Hematocrit, **(B)** plasma glucose (white bars) and lactate (black bars), and **(C)** plasma cortisol (white bars) and skin mucus cortisol (black bars) in rainbow trout after normoxia and after acute hypoxia with different recovery times (1 h, 6 h, and 24 h). The values are presented as mean +SEM (*n* = 8). Significant differences between time points after hypoxia are shown by letters; between hypoxia-treated and normoxia are shown by * (*p* < 0.05) or ** (*p* < 0.001).

Physiological responses were significantly affected by hypoxic stress, and their interaction with time after the challenge was significant for plasma lactate and cortisol levels, in both plasma and skin mucus (Figure 1). In this sense, rainbow trout showed a slight increase in hematocrit 1 and 6 h after suffering acute stress by hypoxia (Figure 1A). Both glucose and lactate levels increased versus control 1 h after hypoxia (+67.4% and +51.8%, respectively), being significant for the latter and showing recovery 6 h after the stressful challenge (Figure 1B). In addition, a significant increase was observed 1 h after stress in plasma and skin mucus cortisol (+207.5% and +786.3%, respectively). Both parameters showed a similar decrease over time, reaching values like those of the control group 6 h after stress (Figure 1C).

**FIGURE 2:**
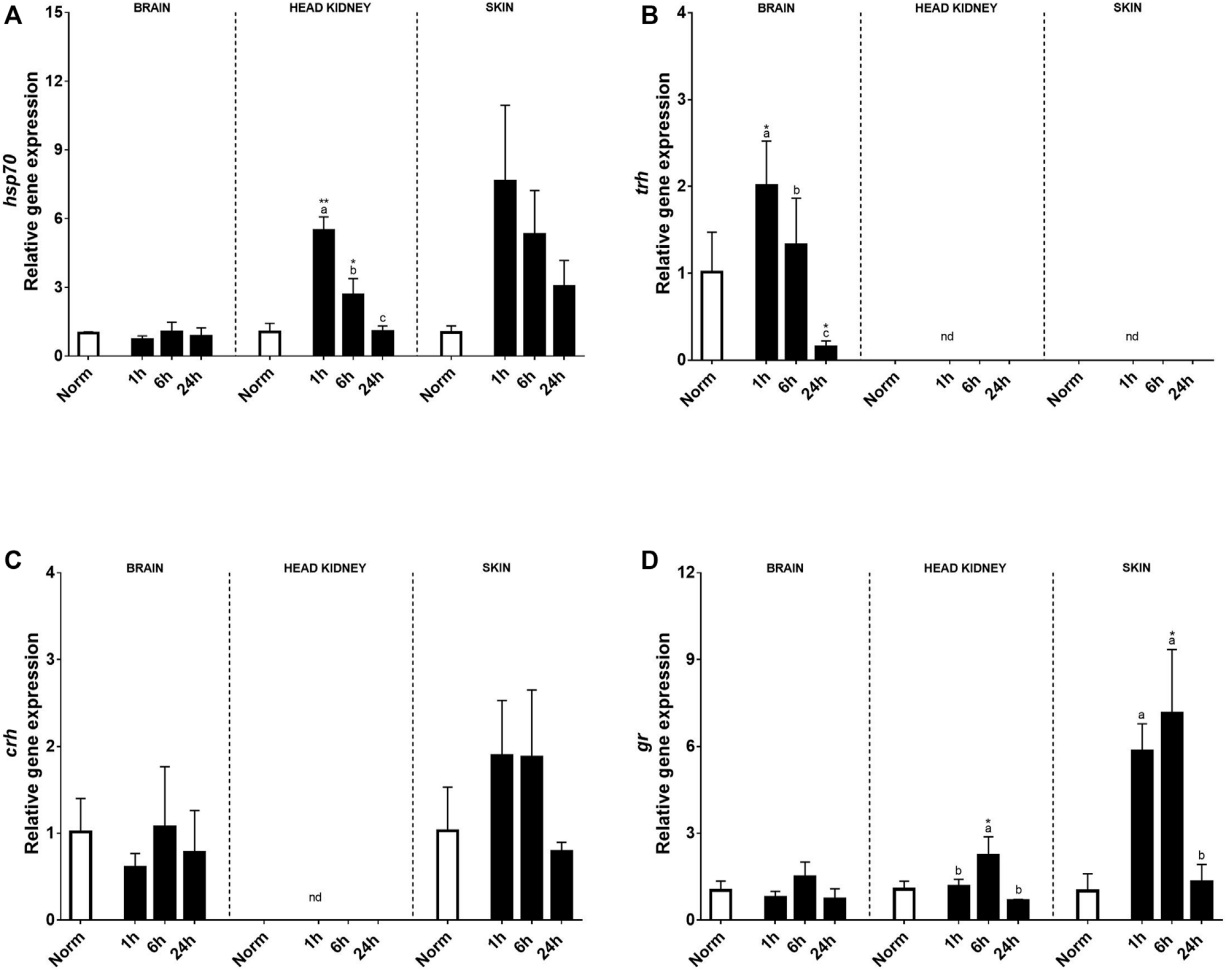
Transcript levels of **(A)** *hsp70*, **(B)** *trh*, **(C)** *crh*, and **(D)** *gr* in rainbow trout brain, head kidney, and skin after normoxia and acute hypoxia with different recovery times (1, 6, and 24 h) (nd: no detected). The values are presented as mean +SEM (*n* = 8). Significant differences between time points after hypoxia are shown by letters; between hypoxia treated and normoxia are shown by * (*p* < 0.05) or ** (*p* < 0.001).

Relative stress-related genes in the brain, HK, and skin of rainbow trout under normoxia and recovery are shown in Figure 2. At the brain level, no effect on *hsp70*, *crh*, or *gr* transcript expression was found by treatment, time, or its interaction, and their expression was low. Instead, *trh* transcript expression significantly increased in the brain 1 h after hypoxia and was downregulated at 24 h compared with that in control fish (Figure 2B). However, a clear effect of acute hypoxia and reoxygenation was detected on the expression of *hsp70* transcripts in HK and skin showing an upregulation in HK 1h and 6 h after acute stress (Figure 2A). Meanwhile, in the peripheral tissue, significant differences due to treatment were found at 1 and 6 h after hypoxia, exhibiting 7.4 and 5.1 times higher relative transcript levels than trout under normoxia, to end up recovering similar values to control at 24 h (Figure 2A). Corticotrophin-releasing hormone expression (*crh*) was not detected in HK, but in the skin, it showed a tendency to increase at 1h and 6 h after hypoxia compared to that in control fish (Figure 2C). Glucocorticoid receptor (*gr*) showed a similar expression pattern in both the brain and HK over time after hypoxia, but significant changes were only found in the later 6 h after acute hypoxia. Moreover, high levels of *gr* transcript expression were detected in the skin, with 5.8 and 7.1 times higher at 1h and 6 h post-stress versus control fish, showing recovery at 24 h (Figure 2D).

**FIGURE 3:**
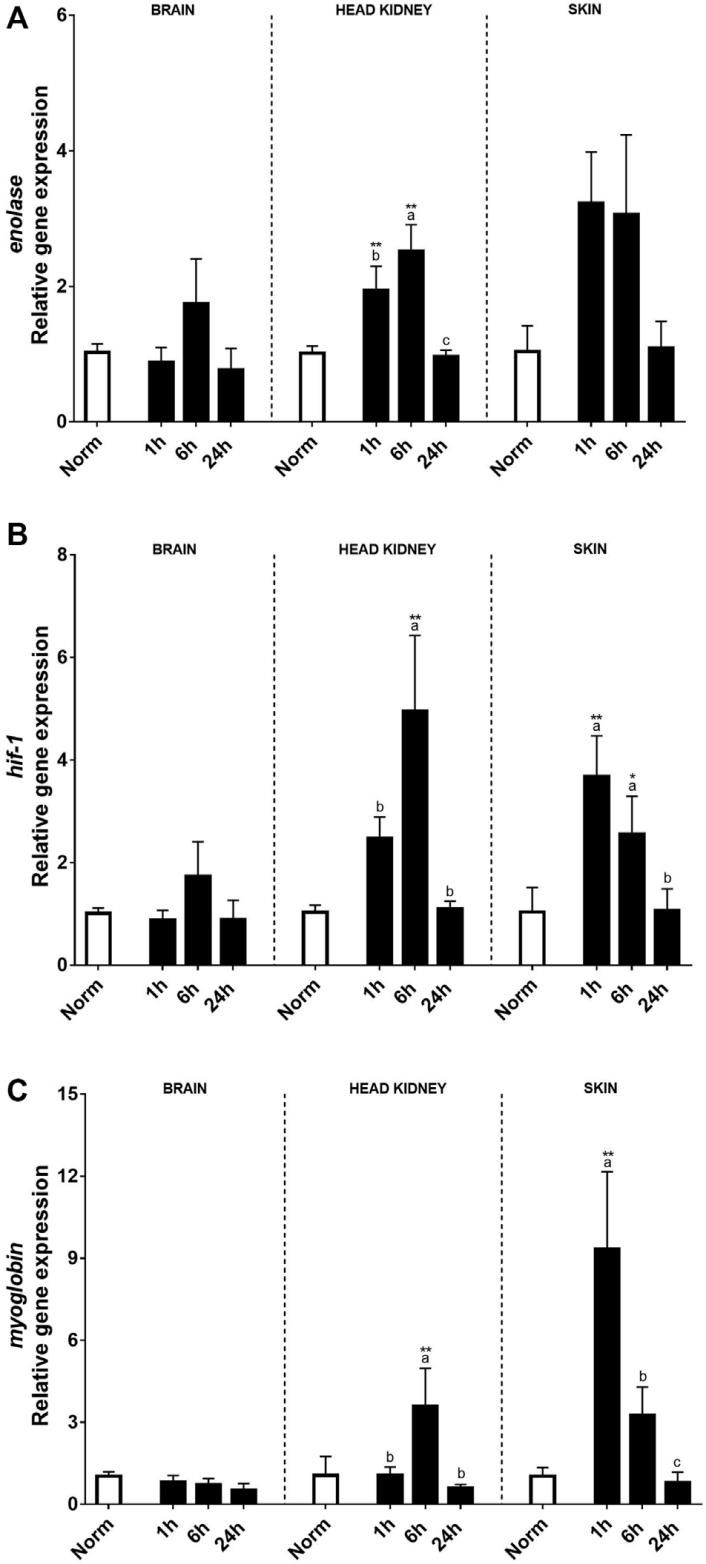
Transcript levels of **(A)** *enolase*, **(B)** *hyf-1*, and **(C)** *myoglobin* in rainbow trout brain, head kidney, and skin after normoxia and after acute hypoxia with different recovery times (1, 6, and 24 h). The values are presented as mean +SEM (*n* = 8). Significant differences between time points after hypoxia are shown by letters; between hypoxia-treated and normoxia are shown by * (*p* < 0.05) or ** (*p* < 0.001).

Hypoxia-related genes including *enolase*, *hif-1*, and *myoglobin* were also evaluated after the hypoxic treatment in the brain, HK, and skin (Figures 3A–C). In this sense, no clear effects after acute hypoxia or during the recovery period were found in the brain, except for *myoglobin* transcript expression that was negatively affected by hypoxia. On the contrary, a clear effect of acute hypoxia treatment was detected in HK and skin for these three genes. *Enolase* transcript expression patterns were similar in HK and skin, rising significantly 1 and 6 h after recovery when compared to the control fish and recovering at 24 h. Despite this, differences between tissues were detected. Thus, in the HK, the highest expression levels were reached 6 h after recovery, whereas in the skin, changes were found 1 h after acute hypoxia. As for *hsp70*, *myoglobin* transcript expression in the skin was very high 1 h after hypoxia, although the values quickly recovered (Figure 3C).

**FIGURE 4:**
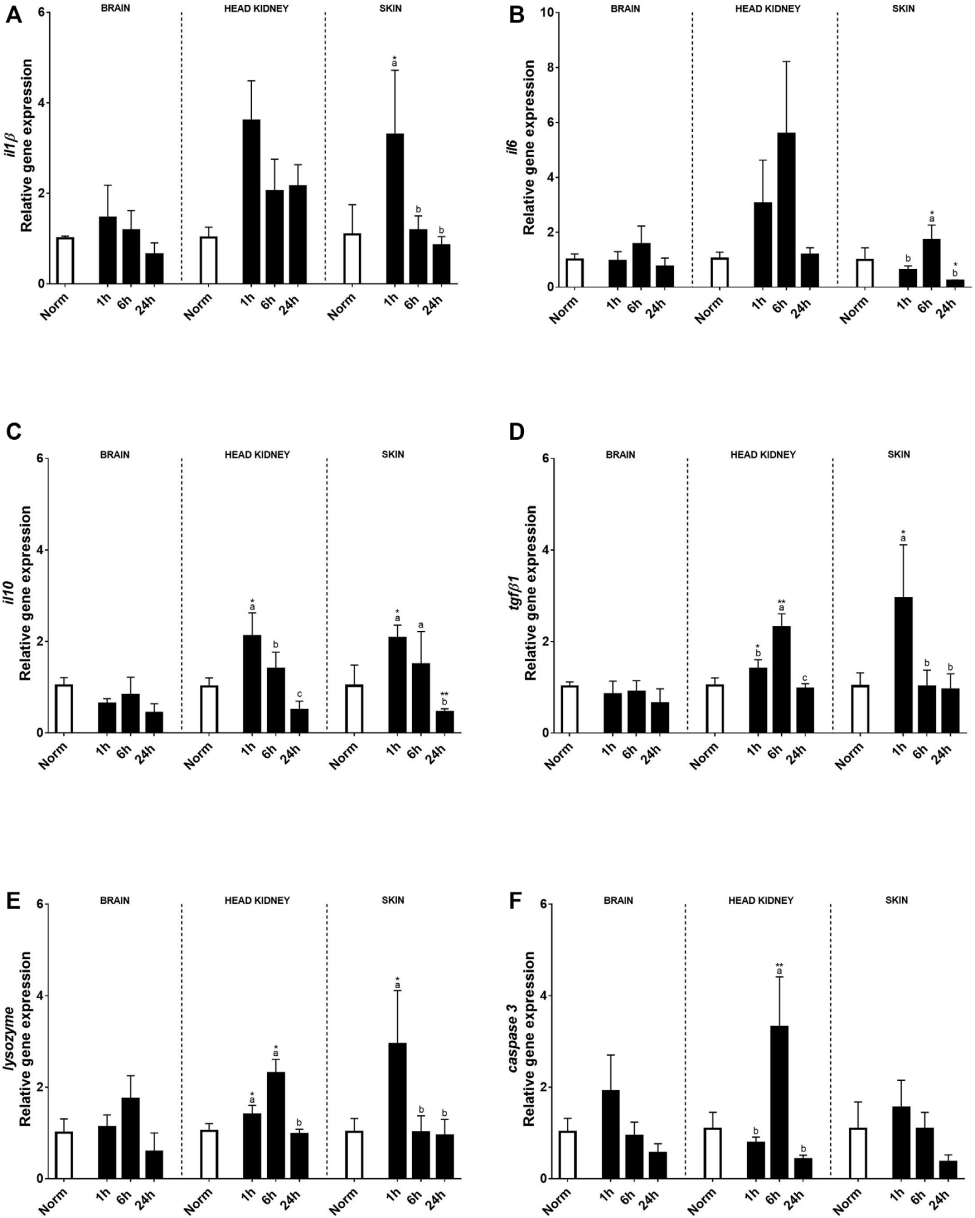
Transcript levels of pro-inflammatory **(A)** *il1β, **(B)** il6*, anti-inflammatory **(C)** *il10* and **(D)** *tgfβ1*, and innate immune response genes **(E)** *lysozyme* and **(F)** *caspase 3* in rainbow trout brain, head kidney, and skin after normoxia and after acute hypoxia with different recovery times (1, 6, and 24 h). The values are presented as mean +SEM (*n* = 8). Significant differences between time points after hypoxia are shown by letters; between hypoxia treated and normoxia are shown by * (*p* < 0.05) or ** (*p* < 0.001).

Also, several immune-related genes were analyzed (Figure 4). In the brain, acute hypoxia did not significantly alter the expression of pro-inflammatory or anti-inflammatory cytokines when compared to the normoxia condition (Figures 4A–D). Despite this, *il10* transcript expression in the brain was affected by hypoxia showing a reduction compared to the control group at 24 h post-stress (Figure 4C). Concerning innate immune key genes, significant changes were found for *lysozyme*, but no effect was detected in *caspase 3* expression (Figures 4E, F). In HK, most genes were affected by acute hypoxia stress, except for *il10* and *caspase3*. On the contrary, in the skin, no effect of hypoxia was detected, but the recovery time and its interaction with treatment were significant. In this sense, both *il1β* transcript expression and *il10* transcript expression rose 1 h after the challenge, recovering basal values for *il10* at 24 h, whereas *il1β* still remained high in HK (Figures 4A, C). However, for *il6* in both tissues and *tgfβ1*in HK, changes were found 6 h after recovery, the expression of the former being only significant in the skin, whereas *tgfβ1* was significantly upregulated 1 h after hypoxia (Figures 4B, D). Moreover, the expression profiles of *lysozyme* and *caspase3* in HK were the same, showing the highest expression 6 h after hypoxia (Figures 4E, F). In the skin, an upregulation of *lysozyme* expression was found 1 h after hypoxia, whereas no effects were found for *caspase 3*.

**FIGURE 5:**
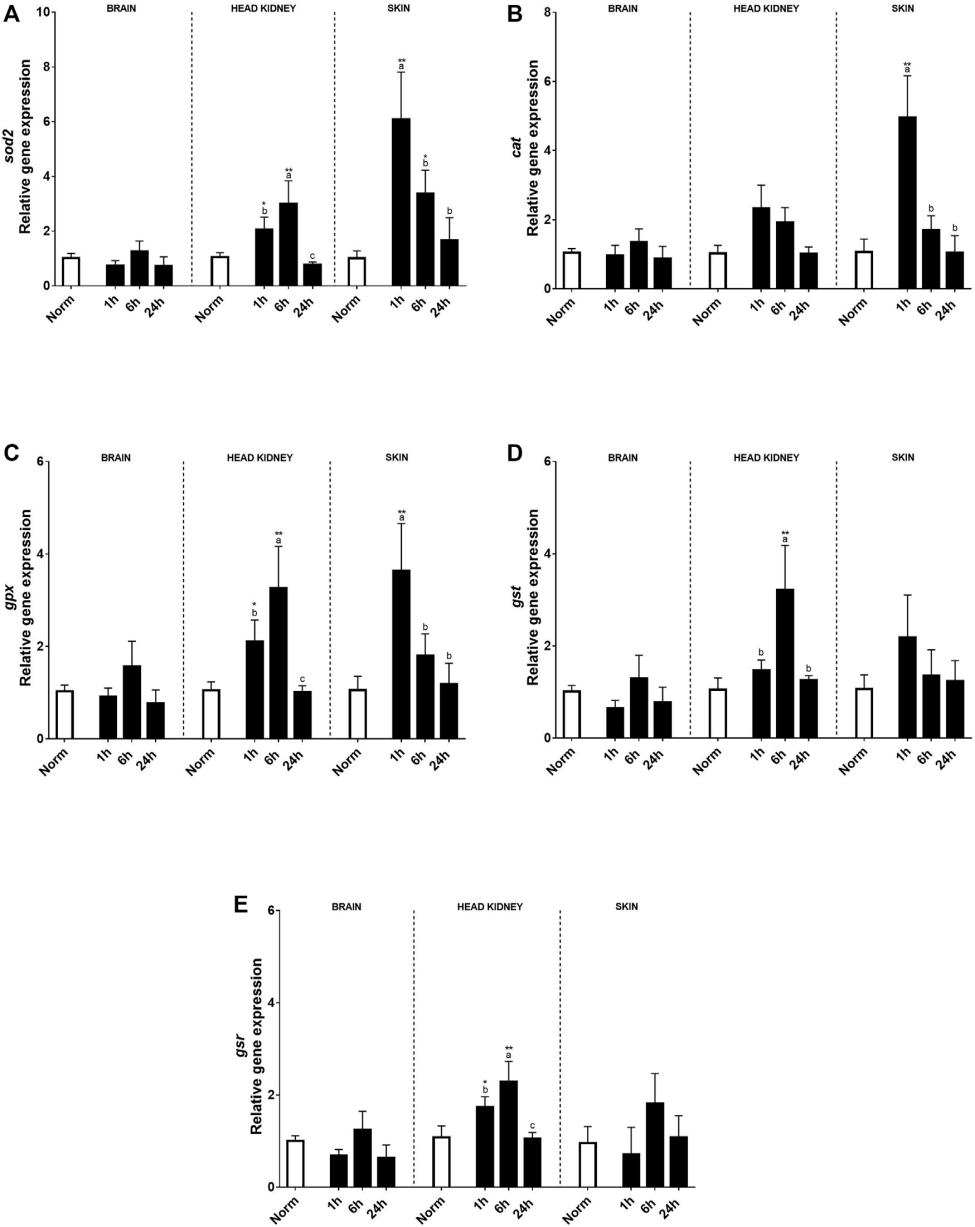
Transcript levels of antioxidants **(A)** *sod2*, **(B)** *cat*, **(C)** *gpx*, **(D)** *gst*, and **(E)** *gsr* in rainbow trout brain, head kidney, and skin after normoxia and acute hypoxia with different recovery times (1, 6, and 24 h). The values are presented as mean +SEM (*n* = 8). Significant differences between time points after hypoxia are shown by letters; between hypoxia treated and normoxia are shown by * (*p* < 0.05) or ** (*p* < 0.001).

No effects on antioxidant transcript expression were found in the brain (Figure 5). Contrary to the brain, in the HK, a clear effect of hypoxia treatment and time (6 h after recovery) was found in most of the genes studied compared to the fish under normoxia. Thus, *sod2*, *gpx*, *gst*, and *gsr* transcript expression increased 1 h after the hypoxic challenge and kept upregulated even more than 6 h after recovery and recovered in similar levels to the control fish at 24 h (Figures 5A,C–E). In the skin, a clear effect of acute hypoxia, time, and its interaction was detected for *sod2*, *cat*, and *gpx. Sod2* that constitutes the first line of defense against oxidative stress upregulated its expression 1 h after hypoxia and slowly decreased over time, showing slight differences with control rainbow trout after 24 h (Figure 5A). On the other hand, *cat* and *gpx*, antioxidants of the second line of defense, showed a peak of expression at 1 h after acute hypoxia and recovered 6 h post-stress (Figures 5B, C).

## Discussion

The present work intended to determine what are the responses of rainbow trout under the experimental circumstance of an acute decrease of dissolved oxygen in the water (down to 2 mg/L), followed by a recovery period of 1, 6, and 24 h. This type of transient episodes may take place in case of a sudden power supply or failure of RAS aeration systems or in nature due to episodes that can occur more frequently due to global warming. Other than previous works associated with air exposure, the present results want to determine the response once the fish has experienced water hypoxia and their alarm systems have been activated and put in place the mechanisms of recovery. Moreover, this work assessed gene transcript levels associated to neuroendocrine and immune systems, oxidative stress, and metabolism in order to provide a wider overview of the recovery process. It should be mentioned that the present study also intends to emphasize the response and the role of skin which is noteworthy in response to biotic and abiotic environmental stressors.

Dissolved oxygen (DO) is a key environmental element for fish, since water has low capacitance and diffusion constant (Richards, 2009). Thus, the reduction in DO involves adaptation of physiological mechanisms to compensate for the lower water extraction and distribution, or fish must reduce their energetic expenditure to match oxygen availability (Abdel-Tawwab et al., 2019; Yoann et al., 2019).

A hypoxic environment thus becomes a significant stressor, so fish trigger stress responses, with the adrenergic responses, increase in respiration rates, and gill ventilation being the first reaction (Hou et al., 2020; Bergstedt et al., 2021). Nevertheless, some fish may use other strategies associated to behavior such as remaining quiet to decrease their metabolic rate or moving upward to the water surface and using transient air breathing (Chapman and McKenzie, 2009; Bowyer et al., 2014; Obirikorang et al., 2020). In the present study, rainbow trout, a hypoxia-sensitive species, would combine both strategies during the hour that were kept in hypoxia at 2 mg O_2_/L, as also suggested by other authors that exposed trout to DO in water below normoxic levels (7 mg/L) (Abdel-Tawwab et al., 2019; Hou et al., 2020). After hypoxia, the non-specific stress response was induced, the HPI axis was activated, and cortisol was released showing significantly higher levels of plasma cortisol 1 h after hypoxia and returning to basal levels after 6 h recovery, which is in agreement with a similar response found in other species (Weendelar-Bonga, 1997; Abdel-Tawwab et al., 2019; Pinedo-Gil et al., 2019). This hormone is considered a highly sensitive signal of stressed fish since the difference between basal and stressed levels is very high (Mommsen et al., 1999; Ellis et al., 2012). A previous work of our team showed that rainbow trout needed longer time to recover basal cortisol levels when the stressor was 1 min of air exposure (Khansari et al., 2019), suggesting that the stressor was stronger for trout, indicating that the type of stressor is a very important factor to consider. Also, plasma glucose and lactate were increased at 1 h sampling time, showing a positive correlation with cortisol and recovering basal values 6 h after hypoxia. Similar results were observed in triploid rainbow trout by Han et al. (2022), in turbot (Jia et al., 2021), and in rainbow trout exposed to acute stress hypoxia (4 mg O_2_/L) and crowding within 10 min (Pinedo-Gil et al., 2019). The absence of time effect on glycemic levels after hypoxia could be due to the fact that the stressor was not intense enough to induce sufficient gluconeogenesis and glycogenolytic action, or to the fact that one of the mediators of such response, catecholamines, shows a very quick response (Weendelar-Bonga, 1997), which did not help to detect significant changes at the sampling time assessed. The increase in plasma lactate indicates the switch from aerobic to anaerobic metabolism, leading to an accumulation of lactate and protons (Wang and Richards, 2011). This generates an oxygen deficit; since during the recovery period, oxygen is needed for the oxidation of anaerobic metabolites and energy store refilling (Bergstedt et al., 2021). In this sense, a lower oxygen deficit is related with a higher hypoxia tolerance (Svendsen et al., 2012; Genz et al., 2013). The recovery of lactate basal levels in rainbow trout after 1 h in hypoxia took place 6 h after stress, pointing out to a rapid clearance of accumulated metabolic by-products and promoting survival avoiding lactic acidosis. Also, an increase in hematocrit was found due to hypoxia treatment that could be due to adrenaline-induced erythrocyte swelling, release of preformed red blood cells by spleen contraction (Lai et al., 2006), changes in plasma volume, plasma skimming, and new red blood cell formation, as described in previous works (Nikinmaa, 1990; Weendelar-Bonga, 1997; Gallaugher and Farrell, 1998; Pichavant et al., 2002; Nikinmaa and Tervonen, 2004; Aboagye and Allen, 2018; Léger et al., 2021).

Regarding the gene expression patterns, it was intended again to assess key genes implicated in stress, immunity, and metabolism. An upregulation in *trh* was found in brain 1 h after hypoxia, followed by a significant downregulation after 24 h. A downregulation has also been previously found over time in the sea bream brain after air exposure suggesting an inhibition of the thyroid hormone axis, preventing unnecessary energy loss (Skrzynska et al., 2018; Liu et al., 2019), and similar results were also found for this species due to salinity changes (Ruiz-Jarabo et al., 2017). In the present study, *crh*, *gr*, or *hsp70* did not show relevant changes at the brain level, suggesting that the kind of hypoxia challenge applied was mild or moderate. Contrarily, *hsp70* and *gr* transcript expression was significantly upregulated in both the HK and skin of rainbow trout. Similar results to those on *hsp70* in the HK were found in the rainbow trout spleen after air exposure (Khansari et al., 2019), suggesting that both tissues are more intensively involved in immunological functions than in other non-specific stress responses. In addition, *gr* mediates the influence of cortisol in modulating gene expression in target tissues post-stress (Vijayan et al., 2003). On the contrary, *hsp70* regulation in the liver, an organ more related to metabolism, presented a different expression pattern after air exposure, showing tissue-specific response after stress (Khansari et al., 2019). Regarding the skin, the transcript-level profile of *hsp70* previously found after air exposure over time by Khansari et al. (2018) was completely different from the one found in the present study, where a significant increase was found even at 24 h after hypoxia. Other studies on common carp (*Cyprinus carpio* L.) skin showed low levels of *crh* expression, like in the present study, suggesting the putative presence of a local cutaneous CRH system. Thus, Mazon et al. (2006) observed CRH-positive cells in the dermis and epidermis of common carp, and it should be mentioned that this cutaneous CRH system is well-established in mammals, as CRH has been demonstrated to be produced by various immune cells in peripheral areas, including macrophages (Baker et al., 2003). So, CRH can been found in several peripheral tissues and may be involved in the regulation of the distribution of immune cells after acute stress (Huising et al., 2003) or infection (Scharsack et al., 2003). This idea was reinforced by the transcript levels found in the skin, since most of them were significantly upregulated just 1 h after recovery, whereas the HK response was found later (in most cases, 6 h after hypoxia) and at lower levels, as for glucocorticoid receptor (*gr*) expression. Thus, the results confirm that the skin plays a significant role in reacting to environmental challenges, and its fast reaction can be attributed to the potential of this tissue to respond to direct contact with the environment.

Stress generates a non-specific and integrated response involving all regulatory systems (neural, endocrine, and immune), and some of the molecules implicated in such a non-specific response are pro-inflammatory cytokines even if the stressor is not a pathogen or a microorganism (Castillo et al., 2009; Khansari et al., 2019). The pro- and anti-inflammatory responses in both the HK and skin followed a similar increment pattern, suggesting the existence of a fine adjustment of these components, since their imbalance may lead to tissue damage and immune dysfunction (Delves et al., 2006; Verburg-van Kemenade et al., 2011). In the skin, transcript level response was faster than the HK, showing a significant increase 1 h after recovery, except for *il6* that reached a maximum of 6 h after hypoxia. Accordingly, high levels of skin mucus cortisol were also found 1 h after recovery, that were also correlated with those found in plasma, reaching basal values 6 h after hypoxia exposure. The results found in the skin are in accordance with a previous experiment in rainbow trout after 1 min under air exposure (Khansari et al., 2018), and the fast upregulation of the genes might be induced by both recirculation and local cortisol secretion. On the contrary, in the HK, most of the gene upregulation takes place 6 h after stress, suggesting that in the peripheral tissue, a local immune response takes place as soon as the stressor is detected, whereas the interrenal tissue participates in a later response and possibly prevents further alteration at the central level by this type of stress, since no differences in the transcript levels were found in the brain. Enolase, a metabolic and energetic marker involved in glycolysis, would act as a heat-shock protein protecting cells after hypoxia by providing faster access to energetic resources (Soengas et al., 1998; Roland et al., 2000; Gracey et al., 2001). Moreover, a clear upregulation was found in the HK and skin, although in the latter, the changes were not significant. These results were in accordance with a previous study on *Sparus aurata* in which a significant upregulation was found in the brain after an *in vivo* LPS challenge (Ribas et al., 2004).

Oxygen homeostasis in mammals is regulated by the hypoxia-inducible factor (*HIF*) gene that affects the use and transport of oxygen by regulating its expression during hypoxia (Nikinmaa and Rees, 2005; Rimoldi et al., 2012; Baptista et al., 2016; Wang M. et al., 2021). In our results, both the HK and skin showed upregulation in *hif-1* transcript levels by hypoxia, since their levels were significantly higher 6 h after recovery in the HK and 1 and 6 h post-hypoxia in the skin. In yellow catfish, hypoxia also induced alteration in *hif-1* expression in the brain, liver, and gills (Wang M. et al., 2021). The main difference that could explain the absence of changes in the brain between that study and ours could be the species and sampling time. Thus, while Wang et al. sampled just after 1 h hypoxia, we sampled 1 h after recovery, that is, after 1 h in hypoxia followed by 1 h in normoxia. Despite this, the transcript level profile found after recovery was similar in both studies.

Stressful factors, like hypoxia, could contribute to the rise of ROS production (Lushchak, 2011; Pascoli et al., 2011; Rahal et al., 2014), as well as the reoxygenation after hypoxia (recovery period) (Bergstedt et al., 2021). Cells have enzymatic and non-enzymatic antioxidants to prevent oxidative damage to proteins, lipids, and nucleic acids avoiding cell death (Halliwell and Gutteridge, 2015; Pinedo-Gil et al., 2019). If the balance between oxidative damage and antioxidants is broken, inflammatory reactions are triggered that can negatively affect fish cells; therefore, the ability to avoid and/or repair cellular damage becomes crucial. In this sense, an upregulation was found in HK 1 and 6 h after hypoxia in *sod2*, *gpx*, *gst*, and *gsr*, whereas a slight increase was observed for *catalase* transcript levels, that could be due to both hypoxia and recovery period under normoxia since their expression was even higher 6 h post-stress. Catalase activity is often related with high levels of oxidative stress (Box et al., 2007; Gutteridge and Halliwell, 2010; Halliwell and Gutteridge, 2015). Thus, the absence of a significant *catalase* transcript level regulation suggests that, despite time and low DO, rainbow trout did not have problems to deal with oxidative stress at the interrenal level. However, in the skin, the antioxidant transcript levels were quickly upregulated (1 h after recovery) for *sod2*, *catalase*, and *gpx*, suggesting that this tissue needs to avoid and/or repair cellular damage caused by ROS arising from reoxygenation, since the skin is in direct contact with water (Bergstedt et al., 2021). Previous studies also found an increase in the SOD activity in the fish liver and intestine after hypoxia–reoxygenation (Lushchak et al., 2005; Lushchak and Bagnyukova, 2007; Sun Y. et al., 2020; Li et al., 2020) and an increment in antioxidant gene expression in the channel catfish skin after *Aeromonas hydrophila* infection (Li et al., 2013), suggesting its possible involvement in defense against pathogens or in skin wound repair (Espinosa-Ruíz and Esteban, 2021).

Similarly, a study on *Schizothorax prenanti* showed that the antioxidant expression response had two differentiated phases after acute hypoxic stress: an initial one with suppressed *sod*, *catalase*, and *gpx* expression and a later recovery one where there was increased expression of *sod*, *catalase*, and *gpx* (Zhao et al., 2020).

On the contrary, Han et al. (2022) found that *sod*, *catalase*, and *gpx* transcript levels in the liver from triploid rainbow trout were downregulated or unchanged after 10 min under hypoxia and crowding. These differences during hypoxia–reoxygenation could be due to species-, tissue-, and time-specificity, thus resulting in different dynamics.

Myoglobin expression was also studied, since its function was commonly associated with oxygen storage and diffusion. This protein is known to accumulate in some vertebrate tissues, such as in the brain of common carp (Helbo et al., 2012; Mannino et al., 2019) and the brain and heart of goldfish (Roesner et al., 2008), which are both hypoxia-tolerant fish. Our results showed a similar transcript level pattern for myoglobin and antioxidants in both the HK and skin. A recent study demonstrated the peroxidase activity of myoglobin, with a wide reductive substrate-specificity and activation by low pH (Mannino et al., 2019). Thus, its activation led to a faster and more efficient cellular response to hypoxia–reoxygenation-induced oxidative stress than the *novo* synthesis of other antioxidants.

Overall, these results suggest that the type of stressor applied (1 h transient hypoxia at 2 mg O_2_/L) had a mild or moderate effect on rainbow trout, since only moderate changes at the central brain level indicate that fish were able to cope with this particular stressor. Unlike the brain, HK and skin were more affected by hypoxia and reoxygenation. Thus, the results support the important role of the HK as an important endocrine and hematopoietic-lymphoid organ in teleostean fish (Gallo and Civinini, 2003; Uribe et al., 2011), particularly after stress situations. For the skin, our results also showed gene upregulation in general terms, indicating a relevant degree of responsiveness of this peripheral tissue. Since the skin is in direct contact with the hypoxic environment, it could act as a sensory system that could first develop a local response, subsequently leading to a systemic neuroendocrine response (Schreck and Tort, 2016).

In conclusion, the present work shows that transient hypoxic episodes in the water–oxygen content may be regulated by fish, including oxygen-sensitive species such as rainbow trout, even under acute oxygen reduction (2 mg/L). The present results show that brain function is not heavily affected and that most of the regulatory processes are carried out by other coordinated organs and structures, mostly the HK and skin. One relevant result of the present work is the important role of the skin as a sensory and responsive organ dealing with environmental variables such as oxygen availability. The present results show both high skin sensitivity and a wide range of responses both in time and change rates, even more in other central structures such as the brain, under this experimental condition. The present results also show that, even though the stressor is a physical/environmental stressor, all components of the biological systems participate in the regulation of the response process and the recovery process, including neuroendocrine, metabolism, and immunity.

## Data Availability

The original contributions presented in the study are included in the article/Supplementary Materials; further inquiries can be directed to the corresponding author.
